# 67-kDa laminin receptor and cGMP induced cancer-selective apoptosis

**DOI:** 10.1186/2050-6511-14-S1-O33

**Published:** 2013-08-29

**Authors:** Motofumi Kumazoe, Hirofumi Tachibana

**Affiliations:** 1 Division of Applied Biological Chemistry, Department of Bioscience and Biotechnology, Faculty of Agriculture, Kyushu University, Fukuoka 812-8581, Japan; 2 Food Functional Design Research Center, Kyushu University 812-8581, Japan

## Background

**Figure 1:**
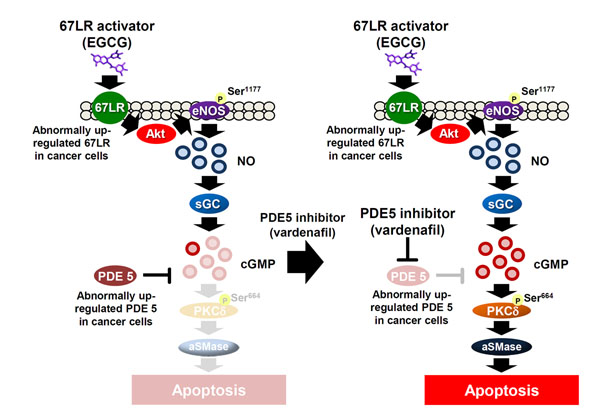
EGCG activates the endothelial nitric oxide synthase (eNOS)/cGMP axis via cancer-overexpressed 67LR (A) EGCG induced eNOS activation through 67LR.

EGCG ((−)-epigallocatechin-3-O-gallate), a polyphenol in green tea, induces apoptotic cell death in cancer cells without affecting normal cells and several clinical trials have been carried out to evaluate its potential value [1,2]. 67-kDa laminin receptor (67LR) has been identified as an EGCG receptor [3]. It has recently been demonstrated that overexpressed 67LR in multiple myeloma (MM) mediates EGCG-induced cancer-specific apoptosis [4-6]. In this study, we revealed that cGMP acts as a cell death mediator of the EGCG-induced anti-MM effect through acid sphingomyelinase (ASM) activation. In this apoptosis pathway, EGCG activated the endothelial nitric oxide synthase (eNOS)/cGMP axis, a well-known mechanism in vascular homeostasis via cancer-overexpressed 67LR. We also demonstrated that cGMP negative regulator, phosphodiesterase 5 (PDE5), was overexpressed in MM cells, and vardenafil, PDE5 inhibitor synergically enhanced the anti-MM effect of EGCG (see Figure 1). This regimen in combination killed MM via overexpressed 67-kDa laminin receptors without affecting normal PBMCs.

## Conclusion

In this study, we demonstrate 67LR activated the peculiar apoptotic signalling eNOS/NO/ cGMP/protein kinase Cδ (PKCδ) pathway. Furthermore, we show the upregulation of cGMP is rate-determining process of this cell death pathway. We demonstrate cancer overexpressed negative regulator of cGMP, PDE5 attenuates the cGMP-dependent cell death induced by EGCG. Vardenafil, one of the PDE5 selective inhibitors used for treating erectile dysfunction potentiates anti-cancer effect of EGCG. These results demonstrate that cGMP elevation caused by targeting the overexpressed 67LR and PDE5 in cancer cells may be a useful approach for cancer-specific chemotherapy.

## References

[B1] Shanafelt TD Call TG Zent CS Leis LF Plant BL Bowen DA Roos M Laumann K Ghosh AK Lesnick C Lee MJ Yang CS Jelinek DF Erlichman C Kay NE Phase 2 trial of daily, oral Polyphenon E in patients with asymptomatic, Rai stage 0 to II chronic lymphocytic leukemia Cancer 2013 119 363 370 10.1002/cncr.27719 22760587 PMC3902473

[B2] Bettuzzi S Brausi M Rizzi F Castagnetti G Peracchia G Corti A Chemo- prevention of human prostate cancer by oral administrationof green tea catechins in volunteers with high-grade prostate intraepithelial neoplasia: a preliminary report from a one-year Proof-of-Principle study Cancer Res 2006 66 1234 1244 10.1158/0008-5472.CAN-05-1145 16424063

[B3] Tachibana H Koga K Fujimura Y Yamada K A receptor for green tea polyphenol EGCG Nat Struct Mol Biol 2004 11 380 381 10.1038/nsmb743 15024383

[B4] Shammas MA Neri P Koley H Batchu PB Bertheau RC Munshi V Prabhala R Fulciniti M Tai Y Steven P Treon SP Goyal RK Anderson CK Munshi NC Specific killing of multiple myeloma cells by (-)-epigallocatechin-3-gallate extracted from green tea: biologic activity and therapeutic implications Blood 2006 108 2804 2810 10.1182/blood-2006-05-022814 16809610 PMC1895573

[B5] Tsukamoto S Hirotsu K Kumazoe M Goto Y Sugihara K Suda T Tsurudome Y Suzuki T Yamashita S Kim Y Huang Y Yamada K Tachibana H Green tea polyphenol EGCG induces lipid-raft clustering and apoptotic cell death by activating protein kinase Cdelta and acid sphingomyelinase through a 67 kDa laminin receptor in multiple myeloma cells Biochem J 2012 443 525 534 10.1042/BJ20111837 22257159

[B6] Kumazoe M Sugihara K Tsukamoto S Huang Y Tsurudome Y Suzuki T Suemasu Y Ueda N Yamashita S Kim Y Yamada K Tachibana H 67-kDa laminin receptor increases cGMP to induce cancer-selective apoptosis J Clin Invest 2013 123 787 799 23348740 10.1172/JCI64768 PMC3561824

